# Supplementary material for: Characterization of Macular Structural and Microvascular Changes in Thalamic Infarction Patients: A Swept-Source Optical Coherence Tomography–Angiography Study
Source: Brain Sci. 2022 Apr 20;12(5):518. doi: 10.3390/brainsci12050518 (PMC9139152; doi:10.3390/brainsci12050518)
Supplement: Supplementary file 1 [file brainsci-12-00518-s001.zip › brainsci-1648303-supplementary.pdf]

Table S1. SS-OCT/SS-OCTA results stratified by duration of thalamic infarction.

	Group1(n=20)	Group 2(n=6)	Group 3(n=9)	*p**	*p* 1	*p* 2	*p* 3
SVP, mm^2	0.198 ± 0.022	0.203 ± 0.022	0.198 ± 0.035	0.502	**0.013**	**0.042**	0.576
ICP, mm^2	0.163 ± 0.026	0.167 ± 0.016	0.175 ± 0.016	0.455	**0.004**	0.200	**0.023**
DCP, mm^2	0.112 ± 0.023	0.118 ± 0.011	0.121 ± 0.024	0.498	0.128	0.980	0.153
RNFL, µm	16.94 ± 1.463	17.704 ± 1.165	17.173 ± 2.315	0.212	0.286	0.138	0.688
GCIPL, µm	61.667 ± 8.131	62.740 ± 5.620	62.161 ± 10.691	0.595	0.211	0.223	0.692

Group 1: < 1 month; Group 2: 1-6 months; Group 3: >6 months.

*p**value among the three groups;

*p*1: Group1 vs Group 2;

*p*2: Group1 vs Group 3;

*p*3: Group2 vs Group 3.

Values in bold indicate $p < 0.05$.
